# Supplementary material for: Griffithsin carrageenan fast dissolving inserts prevent SHIV HSV-2 and HPV infections in vivo
Source: Nat Commun. 2018 Sep 24;9:3881. doi: 10.1038/s41467-018-06349-0 (PMC6155161; doi:10.1038/s41467-018-06349-0)
Supplement: Supplementary file 1 — Supplementary Information [file 41467_2018_6349_MOESM1_ESM.pdf]

Griffithsin/Carrageenan Fast Dissolving Inserts Prevent SHIV HSV-2 and HPV Infections *in vivo*

Derby, et al.

SUPPLEMENTARY INFORMATION

SUPPLEMENTARY FIGURES:

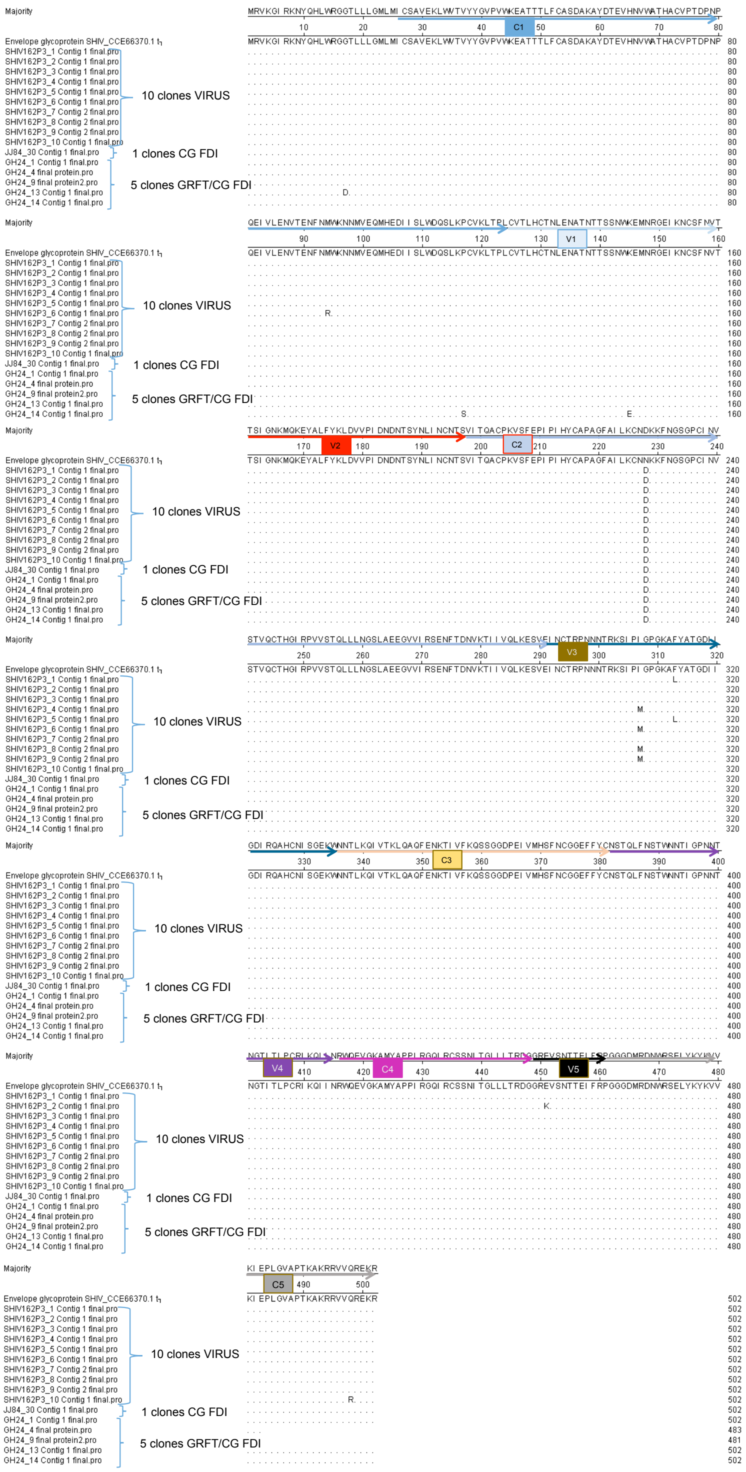

Supplementary Figure 1. Envelope sequencing of SHIV SF162P3 stock and plasma virus in infected macaques. SHIV SF162P3 gp120 sequence was determined by isolating and reverse transcribing viral RNA from the cell-free virus stock and from the plasma of two infected animals as described in Supplementary Methods. cDNA was PCR amplified and cloned into *E. coli* for sequencing. The sequences of SHIV SF162P3 from stock (10 clones) and macaque plasma (5 clones from 1 GRFT/CG FDI animal and 1 clone from 1 CG FDI animal) were aligned against the published sequence of SHIV SF162P3 envelope (Davis, D., Verschoor, E.J. & Fagrouch, Z. Envelope evolution of SHIVsf162p3 in rhesus macaques. in *NCBI GenPept* (2011)). One potential N-linked glycosylation site (PNG, N228) was found to be mutated in the stock virus compared with the published sequence. Variation in the stock centered around the GPGK motif at the tip of the V3 loop and macaque plasma virus lacked the methionine and leucine-containing variants. No variation was detected in the stock virus in regions associated with GRFT resistance and no GRFT-related selection was detected in the few clones obtained from infected animals.

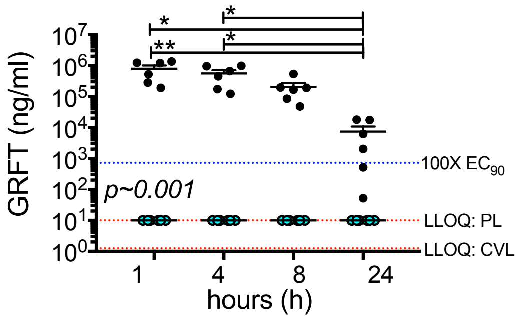

Supplementary Figure 2. GRFT PK profile in macaque CVLs. GRFT levels were measured in non-DMPA-treated macaques 1, 4, 8, or 24 hours post-insertion of GRFT/CG FDIs (n=6 macaques per time point). GRFT concentrations are shown in CVL (black symbols) and plasma (PL, aqua symbols). GRFT was not detected in plasma above the lower limit of quantification (LLOQ) of the assay, which was 10 ng/ml (top red dotted line), and so the values are shown at

the LLOQ. The LLOQ for CVL was 1.25 ng/ml (bottom red dotted line). The 100-fold EC_{90} level, 724.4 ng/ml, is also indicated (blue dotted line). The change in GRFT concentration present in CVL over time was analyzed by Kruskal Wallis test with Dunns post-test for Kruskal Wallis p<0.05. The Kruskal Wallis p value is italicized, and the Dunns comparisons are indicated by asterisks: * <0.05, ** <0.01. For each group, the mean ± standard error of the mean (SEM) is indicated by line and error bars.

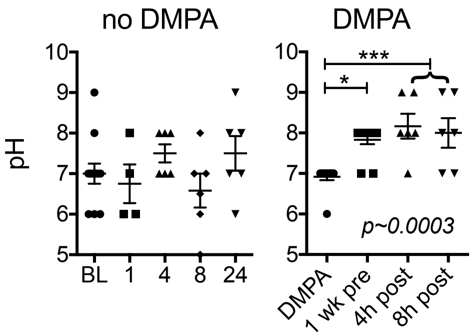

Supplementary Figure 3. Vaginal pH. For non-DMPA-treated macaques (left), the pH was measured at baseline (BL) and at 1, 4, 8, and 24 hours post FDI-insertion (n=6 macaques per time point). For DMPA-treated macaques (right), the pH was measured on the day of DMPA injection (DMPA), one week before FDI insertion (1 wk pre) and then at either 4 hours or 8 hours post insertion (4h post, 8h post, respectively) (n=4-6 macaques per time point). The Kruskal-Wallis test was used to analyze the data, and Dunns pairwise comparisons were performed for a Kruskal-Wallis p<0.05. Kruskal-Wallis p values are italicized and the Dunns comparisons are indicated by asterisks: * <0.05, ***<0.001. 4h and 8h data were combined for comparison of post-FDI with pre-FDI time points in DMPA-treated macaques. For each group, the mean ± SEM is indicated by line and error bars.

SUPPLEMENTARY METHODS

SHIV SF162P3 Envelope Sequencing

The virus envelope was sequenced by a clonal sequencing approach as follows: RNA was isolated from the virus stock using the QIAmp UltraSens Virus kit (Qiagen) as described[61] and reverse transcribed using the Superscript III Reverse Transcriptase with OFM19 5' primer (5'-GCA CTC AAG GCA AGC TTT ATT GAG GCT TA-3') or random oligo hexamers (Invitrogen). PCR amplification of the whole envelope gene (3.2 kb) was carried out using Pfu Taq PCR master mix (Agilent/Stratagene) and the following primers: EnvA (5'-CTT AGG CAT CTC CTA TGG CAG GAA GAA-3') and EnvN (5'-CTG CCA ATC AGG GAA GTA GCC TTG TGT-3'). Nested PCR was carried out on cleaned up PCR products (QIAquick PCR purification kit, Qiagen) using Pfu Taq PCR master mix and the following primers: Env Ph (5'-AAA GAG CAG AAG ACA GTG GCA ATG AGA GTG AAG G-3') and V52 Ph (5'-ATA GTG CTT CCT GCT GCT CCC AAG AA-3') to generate 900 kb products. Amplicons were cloned into *E. coli* (Top10) using the BLUNT TOPO cloning kit (Invitrogen) according to the manufacturer's instructions. DNA was isolated from the colonies (QIAprep Spin Miniprep kit), digested with ECOR1 to check for the presence of the 900 kb insert, and insert-containing samples sequenced (Genewiz) with three primers: V31 (sense strand, 5'-TCA GCA CAG TAC AAT GTA CAC ATG GAA T-3'), AV307 (anti-sense strand, 5'-TCT TCT TCT GCT AGA CTG CCA T-3'), and V32 (anti-sense strand, 5'-AGT AGA AAA ATT CCC CTC CAC AAT TAA A-3'). Sequences were analyzed using DNAStar Lasergene 8.
